# Meanings of nurses’ role in Child and Adolescent Psychosocial Care Centers

**DOI:** 10.1590/0034-7167-2023-0124

**Published:** 2023-12-04

**Authors:** Taliane Machado de Oliveira Leal, Caick Bispo de Souza, Isabela Martins Gabriel, Letícia Giácomo Alexandrin, Aline Cristiane Cavicchioli Okido, Lucía Silva, Diene Monique Carlos

**Affiliations:** I Universidade Federal de São Carlos. São Carlos, São Paulo, Brazil; II Universidade Federal de São Paulo. São Paulo, São Paulo, Brazil

**Keywords:** Child, Adolescent, Mental Health, Nurse’s Role, Qualitative Research, Niño, Adolescente, Salud Mental, Rol de la Enfermera, Investigación Cualitativa, Criança, Adolescente, Saúde Mental, Papel do Profissional de Enfermagem, Pesquisa Qualitativa

## Abstract

**Objective:**

to analyze the meaning attributed to nurses’ role in mental health care in Child and Adolescent Psychosocial Care Centers.

**Methods:**

qualitative research, anchored in the paradigm of complexity. Data collection was carried out through online semi-structured interviews with ten nurses from São Paulo, between March and September 2022, being analyzed thematically.

**Results:**

the diversity and specificity of a child and adolescent mental health clinic, with the need for expanded, territorial and intersectoral care, were unveiled in addition to a fragmented training in the area. There was a need for a deconstruction of being a nurse to make it possible to produce more inclusive and salutogenic practices.

**Final considerations:**

the need for training nurses with adequate knowledge and skills to care for the mental health of children, adolescents and their families is reinforced as well as permanent education of working teams.

## RESUMO

**Objetivo:**

analisar o significado atribuído ao papel do enfermeiro no cuidado à saúde mental no contexto dos Centros de Atenção Psicossocial infantojuvenil.

**Métodos:**

pesquisa com abordagem qualitativa, ancorada no paradigma da complexidade. A coleta de dados foi realizada por meio de entrevistas semiestruturadas *online* com dez enfermeiros de São Paulo, entre março e setembro de 2022, sendo analisadas tematicamente.

**Resultados:**

a diversidade e a especificidade da clínica de saúde mental infantojuvenil, com necessidade de um cuidado ampliado, territorial e intersetorial, foram desveladas além de uma formação fragmentada na área. Houve a necessidade de uma desconstrução do ser enfermeiro para tornar possível a produção de práticas mais inclusivas e salutogênicas.

**Considerações finais:**

reforça-se a necessidade da formação de enfermeiros com conhecimentos e habilidades adequadas para o cuidar da saúde mental de crianças, adolescentes e suas famílias, bem como educação permanente das equipes atuantes.

## RESUMEN

**Objetivo:**

analizar el significado atribuido al papel del enfermero en el cuidado de la salud mental en el contexto de los Centros de Atención Psicosocial del Niño y del Adolescente.

**Métodos:**

investigación con enfoque cualitativo, anclada en el paradigma de la complejidad. La recolección de datos se realizó a través de entrevistas semiestructuradas en línea con diez enfermeros de São Paulo, entre marzo y septiembre de 2022, siendo analizados temáticamente.

**Resultados:**

se develaron la diversidad y especificidad de la clínica de salud mental infanto-juvenil, con la necesidad de una atención ampliada, territorial e intersectorial, además de una formación fragmentada en el área. Existía la necesidad de una deconstrucción del ser enfermero para posibilitar la producción de prácticas más inclusivas y salutogénicas.

**Consideraciones finales:**

se refuerza la necesidad de formación de enfermeros con conocimientos y habilidades adecuados para el cuidado de la salud mental de los niños, adolescentes y sus familias, así como la educación permanente de los equipos de trabajo.

## INTRODUCTION

Since the Psychiatric Reform movement and the child and adolescent mental health policy implementation as an agenda to be met by the Brazilian Health System (SUS - *Sistema Único de Saúde*), there have been developments regarding children’s and adolescents’ mental health care^(1)^. Despite these advances, the look at this population in psychological distress continued to be neglected in practices^(2)^.

Within the scope of Brazilian child and adolescent mental health policies, the creation of Psychosocial Care Centers for children and adolescents (CAPSij - *Centros de Atenção Psicossocial infantojuvenil*) stands out, regulated in 2002. They are health services responsible for the care of children and adolescents in severe psychological and emotional distress as well as commitment, due to the abusive use of alcohol and other drugs and/or situations of relevant psychosocial vulnerability, and must be present in a municipality with more than 70,000 inhabitants^(2)^.

It is worth noting that CAPSij is part of the Psychosocial Care Network (RAPS - *Rede de Atenção Psicossocial*), which proposes to articulate health care strategies in an integral and continuous way, establishing a horizontal link between people and services. This network provided an evolution in the field of children’s and adolescents’ mental health, reducing psychiatric hospital beds and unnecessary hospitalizations, in addition to working as assistance guidance to its users and communities and creating spaces to promote socialization^(3)^.

Despite the field perspective of mental health, in which actions are articulated and developed by the entire interprofessional team, there are practices that should be core to nurses. Nurses constitute a strategic professional at CAPSij, given their proximity to users, reference in essential and specific care, with skill in handling information between users, other team members and network services. In addition, it plays an important role in the elaboration of a Singular Therapeutic Project (PTS - *Projeto Terapêutico Singular*), which favors team integration and decisive actions^(4)^.

The literature has been pointing out challenges for the practice of nurses in this area, especially in performance guided by the psychosocial model and in the exchange of knowledge between professionals who work in CAPS, building actions in the field of mental health beyond nuclear actions^(3-4)^. Another challenging aspect is the understanding, by the nursing team itself, of the restriction in their performance, moving away from the place of those who care and recognizing therapeutic care as the work of the other in CAPSij. It characterizes other team professionals as therapists, and is minimized by the action of the core of nursing^(5)^.

Studies have been based mostly on looking at mental health focused on the adult public, still without bringing the specificities of nursing^(3-4,6)^. Nurses have the skills and abilities to outline comprehensive mental health care for children and adolescents, which can result in expanded assistance that encompasses the individual, family and social contexts^(4)^. Considering the challenges for consolidating nurses’ role in comprehensive mental health care for children and adolescents in a CAPSij, the study question is: how is the construction of nurses’ role in child and adolescent mental health care?

## OBJECTIVE

To analyze the meaning attributed to nurses’ role in mental health care in CAPSij.

## METHODS

### Ethical aspects

The study was conducted in accordance with Resolutions 466/2021 and 510/2016 recommendations on research involving human beings, approved by the Research Ethics Committee of the *Universidade Federal de São Carlos*.

### Study design

This is a qualitative research^(7)^, anchored in the theoretical framework of the complexity paradigm^(8)^. This framework seeks a connection of knowledge characterized by order, disorder and uncertainty. In this way, balance and imbalance, organization and reorganization are established, in a constant process of creation and adjustments^(8)^. Systemic and hologramatic principles will be used as guides. The systemic principle links the knowledge of the parts to the whole, signaling that “the whole is more than the sum of the parts”. The hologramatic principle, in contrast to the systemic principle, indicates that not only the part that is in the whole, but the whole that is equally in the part^(8)^. It is understood that the articulation of these principles will allow looking at the specificity of nurses’ core actions contextualized in the field of child and youth mental health.

To support the construction of this study, the COnsolidated criteria for REporting Qualitative research (COREQ) checklist was used.

### Methodological procedures

#### Study setting

The study settings were CAPSij in the state of São Paulo. Such a choice of specific state is justified by the complex paradigm, which emphasizes the need for constant articulation of data with the context that intertwines them^(8)^. In this way, a certain homogeneity given by the municipalities of São Paulo was sought. According to the Brazilian National Register of Health Establishments (CNES - *Cadastro Nacional de Estabelecimento de Saúde*), in 2022, there were 267 establishments registered as CAPSij distributed across various regions of the state of São Paulo.

#### Data source

The study had 10 nurses as participants, three from the municipality of Campinas, one from Taquaritinga, one from Indaiatuba, one from Piracicaba, and four from São Paulo. Nurses who provided direct assistance to children and adolescents in CAPSij in the state of São Paulo and who had worked in the service for at least one year were included. Nurses who had no contact after five attempts were excluded from the study.

To form the initial sample, the “snowball” recruitment technique was used. Such a strategy makes the researchers characterize the members of their sample and identify a person or a group of people congruent to these characteristics^(9)^. In the case of this study, the first participants were invited from an existing research group at the master’s student’s university and from disclosure in a social network group about mental health. For the indicated participants and those who accepted via social network, an explanatory text was created describing the research objectives. This text was sent by message on the free WhatsApp social network using a personal number chosen by participants. Contacts were made with potential respondents between March and September 2022 by the master’s student. Seven nominated participants were contacted and agreed to participate. From these, six people were indicated for participation, of which three accepted and three refused.

#### Data collection and organization

Data collection was performed through individual semi-structured interviews^(10)^ mediated by free virtual platforms. Prior to the beginning of the interviews, professionals filled out a questionnaire for sociodemographic characterization. Semi-structured interviews started with certain questions, guided by a script, with the following open and guiding questions: what is it like to be a nurse in a CAPSij? What is the role of a CAPSij nurse? What are your actions as nurses in CAPSij? What made it easier and/or difficult for you to act as nurses at CAPSij? During your graduation, did you have specific contact with this public? Do you have any suggestions for training nurses in this area? At the end, a space was also opened to participants for new placements, if they wished to.

After a first approach, we clarified in detail procedures for participating in the research, forwarding the Informed Consent Form (ICF), together with nurse characterization in an electronic form on Google Forms. Subsequently, the interview was scheduled via the online platform, preferably by the interviewee, according to their availability.

Nine interviews were carried out through the free virtual platform Google Meet, and one was carried out through the Zoom platform. All sessions were recorded in audio and image, after agreement by participants in the ICF and authorization to use the image strictly for the purposes of data analysis. The interviews took place from 03/31/2022 to 09/06/2022, with an average of 33’20” (thirty-three minutes and twenty seconds). Respondents were named “Nur”, and numbered in the sequence in which the interviews were conducted. Participating municipalities will not be specified in order to avoid identifying participants.

It should be noted that the number of participants did not seek to achieve only numerical representation, but rather a deepening of the theme as well as the ability to reflect the complexity of the phenomenon in its multiple dimensions^(10)^. Thus, data saturation (indicative elements were discussion of actions, issues related to training and challenges for building the role) occurred in the eighth interview. Two more interviews were carried out, as they were already scheduled and as recommended by the literature, for further validation of the saturation process^(10)^.

#### Data analysis

Data were analyzed using reflective thematic analysis^(11)^, following the steps below: (I) familiarization with the data; (II) encoding, identifying 35 initial codes, grouped into 12 intermediate codes; (III) search for themes, with codes from the previous phase grouped into two themes; (IV) theme review; (V) theme definition and naming; (VI) final writing. For greater data reliability and validity, the construction of initial codes was carried out by two independent researchers, with conflicts resolved by a third one. Interpretive summary was returned to participants for member-checking, with no further additions.

## RESULTS

Participants were mostly women (n=9, 90%), aged 31 to 40 years (n=9, 90%), married or in a stable relationship (n=7, 70%), without children (n =8, 80%), white (n=8, 80%), with about 10 to 15 years of experience as nurses (n=7, 70%) and 2 to 6 years of experience in CAPSij (n=7, 70%). According to the data collected, three nurses had access to content related to child and adolescent mental health during their graduation process. One respondent reported very little contact, through some subjects, such as pediatrics and psychiatry. One respondent reported contact with the subject through conferences and courses after graduation, and one respondent, through specialization courses.

The final themes “Complexity of care” and “Complexity of role” are presented below.

### Theme 1 - Complexity of care

In this first theme, participants highlighted difficulties in working with children and adolescents, as it is a complex, diverse area that requires a specific look, different from caring for the adult public. These issues emerged from the insertion in CAPSij, as it is a clinic with little investment:

*I think that childhood is in limbo, so it seems that they try to frame what the adult production is, and then transfer it to the child one. But childhood and adolescence are another story.* (Nur 2)

[...] *childhood and adolescence are a phase that is largely ignored by health, especially adolescence, they are age groups that have their particularities, right?* (Nur 10)

[...] *the CAPSij clinic is totally different from the adult, you know, because adults end up having their autonomy* [...] *children don’t work like that, we have to pay much more attention to those who are not coming.* (Nur 4)

Nurses also brought the diversity present in the child and adolescent mental health clinic, addressing issues related to syndromes and developmental alterations, even the use of psychoactive substances (UPAS), in addition to the population aged 0 to 18 years being diverse:

[...] *is having to deal with diversity all the time, and more with that look of diversity.* (Nur 2)

[...] *it involves looking from early childhood, from pregnancy to child development, from neurodevelopment to issues of mental health illness, right... so, we work from syndromes, issues of genetic and birth disorders to issues of more biopsychosocial impact, such as substance use among adolescents, for example, depression and anxiety, right?* (Nur 1)

In this regard, for construction of care, they brought the need for a multidimensional look that contemplates an expanded clinic and that considers the family and contexts of life of children or adolescents:

[...] *to think clinically about childhood and youth is to think about where it all begins, it is a way of revisiting childhood that cannot detach itself from social issues, that cannot detach itself from the territory.* (Nur 7)

[...] *care like comprehensive care, how to take care of health and that there is no way to care if we don’t also look at the family, social issues, the school and make this expansion... also working with the shelter, with the assistance network, with the school, with justice, guardianship council*. (Nur 2)

[...] *it is a different logic, which is not about treatment and cure, which is not about illness and medication, it is something else, it is working with other tools, it goes beyond the clinic, it is the whole psychosocial.* (Nur 8)

The nurses interviewed reported the importance of interprofessionality and intersectoriality in the context of children and adolescents, allowing a broader view of health, going through the biopsychosocial elements that are involved in construction of care.

[...] *me and the social worker, we work a lot together in this part of the visit, you know, because, sometimes, we don’t find a solution so much, so we go to the house.* (Nur 3)

[...] *because you have the tutelary council, you have the CMDCA, you have the shelters, so, you go to the case discussion meetings here* [...] *everyone is interested.* (Nur 4)

[...] *multidisciplinary team issues, I think that everyone who is there and who is part of the team, including the people who work at reception, the administrative staff, the people who are there in cleaning, who are there in the pantry, all the professionals who are there are there to provide care and everyone is there to build that care as well* [...] *promoting spaces of care, autonomy, guaranteeing rights, working with the family, working with the school, working with the tutelary council. I think childhood and adolescence have a wealth of devices there that we can work together.* (Nur 8)

They also brought up the importance of these intersectoral actions taking place from the territories where children and adolescents are inserted, which aim at coherence and, in some situations, continuity of care. The nurses interviewed also reported the fragility of academic training in child and adolescent mental health issues. This fragility was due to the absence or small workload allocated to the area or the still biomedical and pathologizing focus. Even when there was *lato sensu* graduate training, they reported that “*I didn’t have any foundation from childhood and we were actually building everything together*” (Nur 3). Empirical experiences through professional experiences are the main generator of learning:

[...] *there is a huge hole in our training* [...] *in my time, we had, wow, the workload of mental health was very little* [...] *I brushed up on some diagnoses, very much based on the disease, the diagnosis, the treatment, in short, in the cure, but I remember it was very little.* (Nur 8)

[...] *the content of specialization was very focused, thinking about adults. I don’t remember, for example, anyone saying that I would behave so I could play with a patient* [...]. (Nur 6)

[...] *non-standard nursing, which we leave formed half square, thinking about a practice that is very focused on the hospital, on interventions that the core sometimes stiffens, crystallizes* [...] *the suggestion is for it not to be so fragmented, for the disciplines to be more interconnected, just as we are called upon to work in a multi-team, thinking about the case in an expanded way.* (Nur 7)

As identified in the last speech, they brought the need for more integrated curricula, both in the area of nursing and in the interprofessional perspective, in addition to the insertion of the theme child and adolescent mental health more intensely, considering the current growing demands of care in the area and by trained professionals.

### Theme 2 - Complexity of role

Based on what was presented in theme 1, nurses identified and reflected on the complexity of building their role in CAPSij. First, they brought a lack of understanding about the service’s objectives to people, including management:

[...] *there is a lot of barrier there in nursing itself, in health management itself, there is no understanding of what the service itself is, what is the demand for this service, what the service receives.* (Nur 3)

[...] *what arises as a need for the service adapts and does it, right, so, it’s challenging.* (Nur 8)

[...] *it’s not clear what the role of each one is, and I think it’s not just the nurse, I think things are getting mixed up... it’s also not so clear what the role is, and there’s something that sometimes I get confused asking, if it is not also convenient that it is not clear?* (Nur 2)

The last lines expose that demands for services are under construction and are dynamic, and that not only the core-field relationship of nursing is not understood, but of other professional areas. This (non)place becomes uncomfortable for the participants, sometimes being placed as a devaluation of the area:

[...] *because I am a nurse, so I see the crossings that people try to make with nursing.* (Nur 6)

[...] *sometimes, we only do pre-consultation, and the rest we support the multidisciplinary team, and the role is different than if it were in an emergency room, in a hospital, you know, it is not well defined like that.* (Nur 9)

Faced with this non-identification of place or role, participants denoted suffering due to the “loss of professional core”, even pointing out that this aspect can weaken the area. The professionals pointed out that, faced with this lack of perception of place, they can take a look at biological issues or support other professionals. Participants unanimously agreed on the need to build a place in child and youth mental health between the core field that goes beyond “standardized” nuclear actions in nursing training to “aggregate and expand”, as reported below:

*You don’t do anything alone, despite having the core north that belongs to your professional scope, but you add* [...] *but, if you expand and work with several professionals* [...]. (Nur 7)

*Nurses cannot be inside a mental health service and want these hospital things inside a mental health service, that’s not how it works, from what I’ve experienced in CAPS.* (Nur 4)

With inherent uncertainties and non-linearities, participants highlighted some possible paths for reflection on the construction of nurses in child and adolescent mental health care. They spoke of a process of deconstruction, including what was learned as a nurse in graduation, to see more inclusive practices, health producers and potentialities in childhood and adolescence:

*So, I consider thinking about mental health already brings this panorama, because it is a deconstruction to get into the mental health clinic, right?* (Nur 7)

[...] *despite saying that we work with health, we are formed to deal with diseases* [...] *to produce health, to be able to see that those human beings, who are in their development process, reach their maximum potential, which is to bring what is healthy that that adolescent has so that they can be in a better condition, to be in the world, I understand that this is what nurses have to do*. (Nur 2)

[...] *but, being able to sit on the floor and play with a child, assess them from another perspective. So, I think there are these different attributions, you know, some very clear, very specific and others that already enter the field of other professions, but which I think is also a role. I think we need to take off our lab coats and understand more about this field of mental health.* (Nur 1)

The movement expressed by Nur 1, of “taking off his coat” and “sitting on the floor and playing with a child”, brings an important meaning to this study in terms of the aforementioned deconstruction. In this context, there is an appeal to legitimize the role, bringing the importance of specific training in the area with theoretical-practical frameworks that instrumentalize the performance in the field of child and youth mental health:

[...] *nursing needs to impose itself and put its foot down a little more and at the same time be extremely technical in what it is going to say so that it is not subjugated, you know so that daily routine does not fall into the merit of thinking that less important, less technical, less scientific* [...] *we have to win the role there and demonstrate why we are here, that it’s not for nothing, that we are at CAPS entrance.* (Nur 6)

[...] *having a different look and, at the same time, being a link between the team, between users and their nursing team, right?* [...] (Nur 5)

Participants listed core nursing actions that need to be valued and recognized in child and adolescent mental health care, such as: the practice of Systematization of Nursing Care (SNC); guidance on medications and routines/life habits; construction of management instruments, such as Standard Operating Procedures, work schedules and supervision of nursing assistants and technicians; and leadership in times of crisis. Finally, a speech brought a summary of what was exposed so far with regard to the complexity of building the role as a nurse working in CAPSij and in child and adolescent mental health care:

*So, you can think of an adolescent who arrives wanting to die, with an idea, after a suicide attempt, or who is doing the cuts* [...] *So, interventions can range from applying a bandage, monitoring the healing process, to what else can you do except cut yourself? Do you have other possibilities? Are there other things that bond with life? What makes life meaningful again? And then, you start thinking about what you expect, you know, with these interventions, reassessing all the time. Within the role of reference, I, as a nurse, manage to move between this more specific issue, thinking about the SNC, and then I am unable to detach the SNC from the Singular Therapeutic Project. I think that, for us, it is even richer, Within the role of reference, I, as a nurse, manage to move between this more specific issue, thinking about the SNC, and then I am unable to detach the SNC from the Singular Therapeutic Project. I think that, for us, it is even richer, because you are able to think more broadly, from much more specific to wider interventions, and, sometimes, to engage in collaboration with other professionals who will assume different clinical settings*. (Nur 7)

## DISCUSSION

The phenomenon studied here, that is, the construction of nurses’ role in CAPSij, is intertwined by elements related to mental health care for children, adolescents and their families, and at the same time to becoming a nurse. Mental health care in CAPSij is, in itself, a complex object, since it encompasses diversity, specificity, non-linearity and standardization, intersectoriality and interprofessionality. These elements coexist with a fragile training in handling such aspects, leading to difficulties in the perception and construction of being a nurse in CAPSij. The whole, understood here by nurses’ role in CAPSij, is reflected in the parts, considered as the elements described, which lead to the difficulty of legitimizing this place. Therefore, this non-place has an impact on the parts that build it, in the emergence of a meaning that needs to be understood in order to indicate possible paths for new constructions^(8)^.

Participant characteristics are similar to a study developed in other contexts, such as age group, female gender, absence of courses in the area of mental health^(4)^. The first category brings the diversity and specificity of child and adolescent mental health care, denoting the need for actions that establish an expanded view of children and adolescents. In that regard, the literature points out that children and adolescents are vulnerable to the effects of trauma and/or stress early in their development process, predisposing to mental health problems. Studies point out that the impacts of external conditions influence the neurodevelopment of this population, contributing to mental illness and may impact the neglect of their own desires and desires^(12-13)^. Thus, corroborating the data from our study, the view constructed for this population needs to consider their development processes and insertions in different life contexts.

It should be pointed out that childhood and adolescence are a peculiar clinic. In Finland, children’s mental health is screened with specific instruments at least once a year, and includes mental health promotion, which is a statutory part of nurses’ work^(14)^. A Swedish study reports that the guiding principles for care in child and adolescent mental health services should contain peer support, empowerment, co-responsibility, based on a unique construction^(15)^. Despite advances in Brazilian public policies, these aspects are still challenges, and mental health care for children and adolescents appears to be focused on illness and little addressed in non-specific contexts, such as Primary Health Care (PHC) services.

In the design of mental health care for children and adolescents, the participants highlighted the need for a systemic and hologrammatic view^(8)^, considering that children and adolescents influence their life contexts and are influenced by them. Thus, a broad view is necessary, especially for families. Studies relate to the importance of families in caring for children and adolescents with mental disorders. A Brazilian cross-sectional study aimed to characterize and compare the importance of involving families of people with mental disorders in nursing care. They demonstrated that nurses’ attitude towards this family involvement in care is essential for a favorable intervention offered to family members. This approach allows expanding the way of working with this population, raising awareness of care and advancing mental health policy^(6)^. Another Chinese study dialogues with Brazilian practice, reporting that mental disorders place greater demands on families, who need greater involvement of health professionals to act and build care that is truly family-centered^(12)^.

In this context of care complexity, the need for intersectoral construction was still present. A Brazilian study reinforces that this direction allows patients to be approached in a comprehensive way, taking into account their different contexts and particularities, which results in a more qualified care. Moreover, it envisages that teams work together and exchange knowledge to ensure adequate and continuous care. Here, care centered on the territories also needs to be highlighted, since it is the broadest community space for user insertion, and the population’s first contact with the health system is often in PHC. “Doing with” is a way of sharing, networking and improving care with effective use of available resources, which is especially important in child and adolescent care^(16)^.

Nurses, as professionals working in child and adolescent care, felt unprepared, reporting in the research a weakness in academic training for their work in the field. A study developed in the state of Santa Catarina, together with nurses working at CAPS II, points to the need for changes in the curricular bases of undergraduate nursing courses, with training focused on the Psychiatric Reform and guaranteeing continuous spaces for permanent education and case studies^(17)^. A study carried out in Ireland with nursing students aimed to assess a role called “clinical tutor” to support learning needs about mental health. A positive experience was demonstrated that favored the articulation of theory concepts with practice as a consequence, establishing a good university relationship with practical nurses, engaging students to work in the mental health clinic^(18)^. Such action can be adapted to the Brazilian context, or improved in places that already occur through the teaching-service integration provided for in the SUS.

Still with regard to aspects of child and adolescent mental health addressed in nursing training, a US study corroborated the need to increase the number of credits in curricular content that address this area. It also exposes the need for qualified places and teachers to teach the content^(19)^. In Brazil, there are still professional practices in services that maintain the asylum logic of care. This can prevent or hinder quality training in mental health for nurses, and it is important that educational institutions and professionals involved seek improvements in these aspects, to ensure that nurses are prepared to act in the RAPS logic and offer quality care to people in distress^(20)^. A dynamic view of training is reiterated, with the need for reformulation of curricula to be more consistent with the reality experienced by people^(19)^. In this regard, there is also a dialogue with the data from this study and the complex paradigm, pointing to the need for more integrated and less fragmented curricula, with mental health being an inherent transversality.

Participants also brought up the need for continuing education on the subject, which is essential for nursing, which has a general education. Corroborating participants’ speeches, authors discussed the existence of a reduction of nurses who worked in child and adolescent mental health as a result of the loss or lack of programs for advanced practice of these professionals^(19)^. There is discussion of qualifying health professionals to deal with mental illness, especially in childhood and adolescence^(21)^. The need for qualification and training for nurses was also highlighted in a literature review, which aimed to outline the gaps in pediatric mental health research^(22)^.

The second category brought reflection on the construction of nurses’ role in CAPSij, which is quite articulated with the complexity of caring for child and adolescent mental health. Due to the fact that nursing education in Brazil still includes a positivist model of understanding/apprehending care and curricula organized into fragmented disciplines^(20)^, it is necessary to deconstruct what is understood as a nurse and nursing to act in this context. Brazilian studies corroborate these challenges, pointing to the need for greater reflection and development of the perception of nurses’ role in mental health, with the aim of improving the quality and efficiency of care provided to users. In addition, these studies highlight the importance of transparency of specific nursing skills in this context^(4)^. These challenges, if not overcome, can become discomfort, difficulties in understanding nursing role and devaluation of the area^(21)^, as pointed out by participants in this study. A study that analyzed the practices developed by nursing professionals in a CAPS II in a large city in Santa Catarina found that, despite advances in nursing care in the biopsychosocial logic, this category still suffers from reductionist attributions of its know-how^(17)^.

It is necessary to recognize the therapeutic potential of psychiatric and mental health nursing, to expand the scope of practice of nurses working in this area. This includes incorporating psychotherapeutic perspectives and interventions, but not displacing other professions. For example, the ability to prescribe and monitor medications by mental health nurses can improve patient safety and provide expanded, high-quality care. Together, this area needs to be reflective, to avoid an uncritical adaptation of the technique and take advantage of the possibilities of applying knowledge and skills in the nursing profession premises^(15)^. An Israeli study denotes cultural competence as fundamental for mental health nurses, indicating the need to strengthen continuous training and the implementation of culturally appropriate care^(23)^. Furthermore, mental health and human rights policies are interconnected and influence each other, and it is necessary to promote both in a positive way, based on ethical values^(24)^.

A scope review highlighted the importance of having a clear understanding and definition of nursing role in the field of psychosocial care, so that nurses can act effectively and contribute to the comprehensiveness of actions. Additionally, support and resources are essential so that these actors can act according to their knowledge and skills, avoiding moral distress and ethical conflicts. It also emphasizes the need for effective communication between the health team and nurses, to ensure that everyone is aligned with health objectives, a fact that enables understanding nurses’ role by the active team^(24)^. We reiterate the importance of permanent education and the implementation of action protocols to support professionals to understand their specificities and roles in an interdisciplinary team. According to a study carried out in Salvador, Bahia, there are still difficulties in collaborative teamwork, especially denoted by the essentially uniprofessional nature of health training. This aspect can lead to the construction of rigid professional identities, with difficulties in team communication and, consequently, restrict expanded care^(25)^.

In this respect, the dialogue between systemic and hologramatic principles^(8)^ is highlighted with regard to the field-core relationship in child and adolescent mental health care, unveiled in the data of this research. It was understood that the emergence of being a nurse in this context is more and less than the sum of core competencies and skills and those acquired in the field of mental health. The emerging phenomenon is dynamic, fluid, unstable and under construction, intertwined by various elements from the social sciences, humanities and nursing. Finding balance in this transit of nuclear and field actions, making use of concepts from the area of mental, collective and child/adolescent health, is seen as a path to this definition.

### Study limitations

This study has limitations. The first relates to the participant recruitment strategy. During the pandemic period, there were difficulties with more directive and on-site searches, which may have led to a more limited number of interested parties. The second is in the virtual technique of data collection, because, despite technological advances, it can hinder the perception of non-verbal communication, relevant for qualitative approaches. Furthermore, although a central concept in the theoretical framework is contextualization, the choice of only one federal state in Brazil may have limited the results, bringing unique realities of this context.

### Contributions to nursing and health

Despite the limitations, this present study’s objective was achieved and has implications for nursing and health practice, namely: (1) due to the area’s complexity and specificity, discussing the need to create action protocols and permanent education for nurses to care in CAPSij; (2) understand specific nuclear and field skills and competences to care for children, adolescents and their families in psychological distress by the team working in CAPSij; (3) develop expanded care for children and adolescents, centered on themselves, their families and their life territories; (4) develop actions that value and promote interprofessional and intersectoral collaboration in child and adolescent mental health care, focusing on the practical and effective construction of RAPS; (5) expand concepts to look at children’s mental health in academic nursing training, considering the transversality of themes and curricular integration, in order to promote preparation and advanced practice in the area.

New studies that deepen the construction of actions between the field and the core of nurses working in child and adolescent mental health are recommended as well as considering the perceptions of nursing professionals who work in PHC and in hospital environments.

## FINAL CONSIDERATIONS

The meaning attributed to nurses’ role in child and adolescent mental health care in CAPSij intertwines elements related to the mental health care of children, adolescents and their families, and, at the same time, to becoming a nurse. It was revealed that the complexity of care in this area is a preponderant element for the construction of this role. Child and adolescent mental health is a complex and diverse clinic, with the need to delineate an expanded, territorial, intersectoral and interprofessional care. These elements led to a misunderstanding of nurses’ place in CAPSij, especially due to the necessary transit between the nucleus and the field for the construction of actions. It brought the need for a deconstruction of being a nurse, to be able to produce more inclusive and salutogenic practices and legitimacy of their role in this space.

This study accessed an audience and a topic rarely addressed in nursing, with a framework that allowed for the broader view it deserves. It reinforces the need to train nurses with adequate knowledge and skills to take care of the mental health of children, adolescents and families. Moreover, there is an urgent need for permanent education for teams working in CAPSij with management support.
